# MicroRNA-181 serves an oncogenic role in breast cancer via the inhibition of SPRY4

**DOI:** 10.3892/mmr.2022.12673

**Published:** 2022-03-04

**Authors:** Yifu Tian, Xiaodan Fu, Qingling Li, Ying Wang, Dan Fan, Qin Zhou, Weilu Kuang, Liangfang Shen

Mol Med Rep 18: 5603–5613, 2018; DOI: 10.3892/mmr.2018.9572

Following the publication of this paper, it was drawn to the Editors’ attention by a concerned reader that the Transwell cell migration assay data shown in Figs. 3B and 9B were strikingly similar to data appearing in different form in other articles by different authors. Owing to the fact that the contentious data in the above article had already been published elsewhere, or were already under consideration for publication, prior to its submission to *Molecular Medicine Reports*, the Editor has decided that this paper should be retracted from the Journal. After having been in contact with the authors, they agreed with the decision to retract the paper. The Editor apologizes to the readership for any inconvenience caused.

